# Non-Suicidal Self-Injury in Adolescents With Major Depressive Disorder: The Role of Beta-Endorphin, Psychological Pain, and Anhedonia

**DOI:** 10.31083/AP52361

**Published:** 2026-08-12

**Authors:** Rabia Sevcan Karaaslan, Oğuz Bilal Karakuş, Nergis Eyüpoğlu, İbrahim Adak

**Affiliations:** ^1^Department of Child and Adolescent Psychiatry, Faculty of Medicine, University of Health Sciences, Van Training and Research Hospital, 65300 Van, Türkiye; ^2^Department of Child and Adolescent Psychiatry, Faculty of Medicine, Trabzon University, Kanuni Training and Research Hospital, 61250 Trabzon, Türkiye; ^3^Department of Child and Adolescent Psychiatry, Faculty of Medicine, University of Health Sciences, Erenköy Mental and Nervous Diseases Training and Research Hospital, 34736 Istanbul, Türkiye

**Keywords:** major depressive disorder, non-suicidal self-injury, beta-endorphin, psychological pain, anhedonia

## Abstract

**Background:**

A substantial proportion of adolescents with major depressive disorder (MDD) engage in non-suicidal self-injury (NSSI); however, the biological and psychological factors differentiating those with and without NSSI remain poorly characterized. This study aimed to investigate whether serum beta-endorphin levels, psychological pain, and anhedonia differ between these groups and to explore their associations with the presence of NSSI.

**Methods:**

The study sample comprised adolescents prospectively recruited during the study period and diagnosed with MDD according to the *Diagnostic and Statistical Manual of Mental Disorders, Fifth Edition, Text Revision* (DSM-5-TR) criteria. Participants were categorized into adolescents with NSSI (NSSI+) and those without NSSI (NSSI−). Assessments included the Children’s Depression Inventory (CDI), Psychache Scale (PAS), and Snaith-Hamilton Pleasure Scale (SHAPS). Serum beta-endorphin levels were measured using enzyme-linked immunosorbent assay (ELISA) from blood samples collected after an overnight fast.

**Results:**

The study cohort consisted of 104 adolescents with MDD, including 52 participants in the NSSI+ group and 52 in the NSSI− group. The sample comprised 85 females (81.7%) and 19 males (18.3%). Compared with the NSSI− group, adolescents in the NSSI+ group demonstrated significantly lower serum beta-endorphin levels and significantly higher levels of depressive symptoms, psychological pain, and anhedonia (all *p* < 0.05). In multivariable analyses, greater depression severity, higher psychological pain, and lower beta-endorphin levels were independently associated with the presence of NSSI. Exploratory mediation analyses suggested an indirect association between depression severity and NSSI through psychological pain.

**Conclusions:**

These findings highlight the relevance of both psychological and neurobiological factors in NSSI among adolescents with MDD and suggest that an integrated assessment of these factors may help identify adolescents at increased risk of this form of self-harm.

## Main Points

1. Adolescents with MDD who engage in NSSI exhibit higher levels of psychological pain and anhedonia, as well as lower serum beta-endorphin levels, compared with those who do not engage in NSSI.

2. In adolescents with MDD, NSSI is independently associated with greater depressive symptom severity, higher psychological pain, and lower serum beta-endorphin levels, but not with anhedonia.

3. Psychological pain may partially account for the association between depressive symptom severity and NSSI, highlighting its potential clinical relevance beyond depressive symptoms alone.

4. These findings suggest that psychological pain and beta-endorphin may contribute to understanding NSSI behavior among adolescents with MDD.

## 1. Introduction

Major depressive disorder (MDD) is a prevalent psychiatric condition characterized by persistent depressive or irritable mood, anhedonia, and functional impairment, often following a recurrent course [1]. In adolescence, the global point prevalence of MDD is estimated to be approximately 8% [2]. Non-suicidal self-injury (NSSI) behaviors, defined as the deliberate and socially non-sanctioned destruction of bodily tissue without suicidal intent, frequently co-occur with MDD [3]. Longitudinal studies have consistently documented a bidirectional relationship between depression and NSSI, whereby depressive symptoms increase vulnerability to self-injurious behavior, and engagement in NSSI contributes to a more severe and persistent depressive course [4,5]. Rates of NSSI among adolescents with MDD have been reported to be as high as 76% in some clinical samples [6]. Adolescents with comorbid MDD and NSSI represent a particularly high-risk subgroup, often demonstrating poorer treatment response and greater clinical burden compared to those with MDD alone [7].

The endogenous opioid system has been a focus of increasing attention as a biological system implicated in both depression and self-injurious behavior [8,9]. Beta-endorphin, a key endogenous opioid peptide, plays a central role in stress regulation, affective processing, and reward functioning through its interactions with dopaminergic and serotonergic pathways [10]. The opioid deficiency hypothesis posits that individuals who engage in NSSI exhibit chronically reduced endogenous opioid activity and may behave in a self-injurious manner to transiently restore endogenous opioid levels [11]. Consistent with this view, reduced beta-endorphin levels have been observed in the cerebrospinal fluid and serum of individuals engaging in NSSI [12,13].

In addition to the neurobiological basis of NSSI, psychological pain, also known as psychache, has emerged as a key construct for understanding self-injurious behaviors. Psychological pain refers to an intense subjective experience of inner distress, perceived inadequacy, or emotional suffering [14]. It has been strongly linked to suicidal ideation and behavior and is consistently elevated in individuals with depressive disorders [15,16]. Current evidence suggests that heightened levels of psychological pain may motivate self-injury as a maladaptive strategy for emotional regulation, particularly during periods of intense affective distress [17].

Anhedonia involves reduced pleasure and loss of interest in usual activities [18], and is a common core symptom observed among adolescents with MDD [19], having been linked to greater illness severity and higher suicide risk [20]. As noted above, it has been suggested that NSSI functions as a means of emotional regulation [21], suppressing negative emotions like numbness and emptiness, and replacing them with positive feelings such as relief and calm [22]. These results suggest that NSSI may be reinforced by its capacity to reduce distress and elicit positive emotional states. Despite the theoretical relevance of anhedonia to NSSI and emerging empirical research examining their relationship, the specific role of anhedonia in NSSI among adolescents with MDD remains insufficiently understood [23].

A growing body of evidence suggests that endogenous opioid activity may be associated with both psychological pain and anhedonia. Psychological pain has been linked to neural pathways that overlap with those involved in physical pain processing, including brain regions associated with endogenous opioid system activity [24]. Similarly, in addition to its association with dopaminergic dysfunction, the endogenous opioid system has been implicated in reward processing and anhedonia [25].

While there is accumulating evidence implicating beta-endorphin dysfunction, psychological pain, and anhedonia in both MDD and NSSI, the extent to which these factors differentiate adolescents with MDD who engage in NSSI from those who do not remains unclear. In particular, there have been few studies jointly examining biological and psychological dimensions associated with NSSI specifically in adolescents with MDD. In light of their relevance to self-harm and MDD-related symptoms, serum beta-endorphin levels, psychological pain, and anhedonia were selected as core study variables that may represent key biologically and clinically related dimensions relevant to NSSI incidence among adolescents with MDD. Although a wide range of factors have been associated with NSSI, the present study focused on beta-endorphin, psychological pain, and anhedonia because these variables may represent theoretically distinct yet potentially interconnected dimensions relevant to both MDD and NSSI, including endogenous opioid functioning, subjective emotional distress, and reward-related dysfunction. In addition, evidence suggests that psychological pain and anhedonia may overlap with neural pathways involving the endogenous opioid system [24,25]. However, relatively few studies have examined these dimensions together among adolescents with MDD with and without NSSI. No prespecified hierarchical relationship among these variables was assumed. Therefore, the present study was developed with the goal of comparing serum beta-endorphin levels, psychological pain, and anhedonia between adolescents with MDD with and without NSSI, and to examine whether these variables were associated with engagement in NSSI among adolescents with MDD.

We hypothesized that adolescents with MDD and NSSI would exhibit lower serum beta-endorphin levels and higher levels of psychological pain and anhedonia relative to those without NSSI. We further hypothesized that beta-endorphin levels, psychological pain, and anhedonia would be independently associated with the presence of NSSI. Given the proposed role of psychological pain in self-injurious behavior and affect regulation, an exploratory mediation analysis was additionally conducted to clarify whether psychological pain may act as an intermediary factor in the relationship between depressive symptoms and NSSI incidence.

## 2. Materials and Methods

### 2.1 Participants

This single-center, case-control study was conducted in the Department of Child and Adolescent Psychiatry of a tertiary mental health hospital between May 2024 and September 2024. The study was planned with balanced group sizes, and adolescents aged 12–18 years who met *Diagnostic and Statistical Manual of Mental Disorders, Fifth Edition, Text Revision* (DSM-5-TR) criteria for MDD were recruited during the same study period. Participants with and without a history of NSSI were included in order to establish balanced study groups. No formal individual matching procedure was performed.

A priori sample size estimation was performed using G*Power version 3.1.9.7 (Heinrich Heine University Düsseldorf, Düsseldorf, Germany). Based on previous literature examining beta-endorphin levels in adolescents with NSSI [26], a medium effect size (d = 0.50), a statistical power of 80%, and a two-sided alpha level of 0.05 were assumed. The minimum required sample size was calculated as 102 participants (51 per group). A total of 104 participants (52 per group) were ultimately included.

During the recruitment period, 153 adolescents diagnosed with MDD were assessed for eligibility. A total of 7 patients were excluded because their depressive symptoms were in remission, 12 were excluded because they had subthreshold NSSI, 17 were excluded due to comorbid psychiatric disorders, 7 did not complete the study assessments and/or blood sampling procedures, and 6 declined participation. The final sample consisted of 104 adolescents with MDD. In accordance with the planned case-control design, the final analysis included 52 adolescents with NSSI and 52 without NSSI.

Psychiatric diagnoses were established using the Turkish version of the Kiddie Schedule for Affective Disorders and Schizophrenia for School-Age Children-Present and Lifetime Version (K-SADS-PL-T). Those participants meeting the DSM-5 criteria for NSSI, defined as engagement in at least five NSSI episodes within the previous 12 months, were classified into the adolescents with NSSI (NSSI+) groups. The remaining enrolled adolescents with MDD who did not meet DSM-5 criteria for NSSI based on clinical interview and K-SADS-PL-T assessment were assigned to the NSSI group. Participants reporting subthreshold self-injurious behaviors that did not fulfill DSM-5 diagnostic criteria for NSSI were excluded from both study groups. The presence or absence of NSSI was determined through DSM-5-based clinical interviews conducted by a child and adolescent psychiatrist and supported by the K-SADS-PL-T assessment. No formal effort was made to evaluate inter-rater reliability with respect to the clinical assessment of NSSI.

Exclusion criteria included the presence of any psychiatric disorder other than MDD, current or past alcohol or substance use disorders, and chronic neurological, metabolic, or endocrine diseases.

### 2.2 Measurements

Data were collected using a structured assessment protocol that included clinician-administered diagnostic interviews and self-report questionnaires. Sociodemographic and clinical information were obtained for all participants. Information regarding current psychotropic medication use and psychotherapy status was also obtained during the clinical assessment. Psychiatric diagnoses were established using the K-SADS-PL-T, and all participants completed standardized self-report measures assessing depressive symptoms, psychological pain, and anhedonia. The presence of NSSI was determined through clinical evaluation based on DSM-5 criteria, and the Inventory of Statements About Self-Injury (ISAS) tool was administered only to participants in the NSSI+ group as a means of characterizing self-injurious behaviors. Body mass index (BMI) was calculated for all participants based on measured height and weight at the time of assessment.

Participants were evaluated with the following standardized instruments:

#### 2.2.1 Kiddie Schedule for Affective Disorders and Schizophrenia for School-Age Children-Present and Lifetime Version (K-SADS-PL)

The K-SADS-PL is a semi-structured diagnostic interview developed by Kaufman et al. [27] to assess current and lifetime psychiatric disorders in children and adolescents. The Turkish version of the scale has demonstrated satisfactory reliability and validity [28]. In the present study, the K-SADS-PL was used to confirm the diagnosis of MDD in study subjects and to identify comorbid psychiatric conditions, which constituted exclusion criteria.

#### 2.2.2 Children’s Depression Inventory (CDI)

Depressive symptom severity was assessed using the CDI, which is a widely used self-report measure developed by Kovacs (1981) [29]. The Turkish version of the scale has demonstrated good psychometric properties in prior reports [30].

#### 2.2.3 Psychache Scale (PAS)

The PAS is a 13-item self-report measure developed by Holden et al. (2001) [31] to assess psychological pain based on Shneidman’s conceptual framework, with higher scores on the scale indicating greater levels of psychological pain. The Turkish version of the scale has demonstrated satisfactory reliability and validity [32].

#### 2.2.4 Snaith-Hamilton Pleasure Scale (SHAPS)

The 14-item SHAPS self-report scale was designed by Snaith et al. [33] to assess state anhedonia. Higher scores reflect greater severity of anhedonia. The scale has been validated and found to be reliable in Turkish [34].

#### 2.2.5 Inventory of Statements About Self Injury (ISAS)

The ISAS, developed by Klonsky and Glenn (2009) [35], is a self-report tool designed to assess NSSI. The scale consists of two parts: Behaviors and Functions. For the current study, the Behaviors section of the ISAS was used to assess the frequency and methods of NSSI. The Turkish version of this tool has previously demonstrated adequate validity and reliability [36].

### 2.3 Blood Collection

Following psychiatric interviews, blood samples were collected via venipuncture from the antecubital vein under sterile conditions between 8:00 and 9:00 AM after an overnight fast by trained medical personnel. Samples were kept at room temperature for one hour to allow for clot formation and subsequently centrifuged at 1000 ×g for 20 minutes at 2–8 °C to obtain serum. The separated serum samples were stored at −80 °C until analysis.

Serum beta-endorphin levels were measured using a commercially available Human β-EP ELISA Kit (Catalog No: E-EL-H0572; Elabscience, Houston, TX, USA) in accordance with the manufacturer’s instructions. Briefly, serum samples were thawed to room temperature, added to pre-coated 96-well plates, and processed according to the standard protocol. Absorbance was measured at 450 nm using a microplate reader.

### 2.4 Statistical Analysis

All statistical analyses were performed using RStudio (R v4.3.0; R Core Team, 2023). Descriptive statistics are presented as mean ± standard deviation (SD) for continuous variables and as frequencies and percentages for categorical variables. The normality of continuous variables was evaluated with the Kolmogorov–Smirnov test.

Group differences for clinical and biological variables of interest (CDI, PAS, SHAPS, and beta-endorphin levels) were initially examined using multivariate analyses of variance (MANOVAs), followed by univariate analyses. Pillai’s Trace was used to evaluate the overall multivariate effect. Group differences for other continuous variables were examined using independent-samples *t*-tests, while categorical variables were compared using the chi-square test. Multivariable logistic regression analysis was conducted to identify factors independently associated with the presence of NSSI, with results reported as odds ratios (ORs) and 95% confidence intervals (CIs). Multicollinearity among predictors was assessed using the variance inflation factor (VIF) values and tolerance statistics.

Exploratory mediation analyses were performed in the full sample cohort (n = 104) using a bootstrap resampling approach with 5000 iterations as a means of estimating indirect effects. Bias-corrected 95% confidence intervals were computed to assess the significance of indirect pathways, providing robust estimates without relying on normality assumptions [37]. In light of the simultaneous measurement of all study variables, these analyses were considered exploratory and hypothesis-generating.

Given the exploratory nature of the study and the limited number of predefined, hypothesis-driven comparisons, no formal adjustment was made to control for multiple comparisons. All statistical tests were two-tailed, and a *p*-value < 0.05 was considered statistically significant.

## 3. Results

A total of 104 adolescents with MDD were included in the study, of whom 52 were classified as NSSI+ and 52 as adolescents without NSSI (NSSI−). No significant differences between these two groups were noted in terms of age, gender distribution, body mass index, family structure, family income level, or family history of psychiatric illness, current psychotropic medication use, ongoing psychotherapy status, or medical comorbidity (all *p* > 0.05; Table 1).

**Table 1.** **Sociodemographic and clinical characteristics of the study groups (n = 104)**.

Variables		NSSI+ (n = 52), M ± SD, n (%)	NSSI− (n = 52), M ± SD, n (%)	Statistics	*p*-value	Effect size
Age (years)		15.84 ± 1.22	15.82 ± 1.50	*t _(102)_* = 0.05	0.960	*Cohen’s d* = 0.010
Gender (f:m)		44:8	41:11	*χ^2^_(1)_* = 0.58	0.446	*Cramer’s V *= 0.075
BMI		22.10 *± *2.60	21.52 ± 3.01	*t _(102)_* = 1.09	0.280	*Cohen’s d *= 0.210
Family structure (nuclear)		38 (73.1)	33 (63.5)	*χ^2^_(1)_* = 1.11	0.292	*Cramer’s V *= 0.103
Current psychotropic medication use		30 (57.7)	27 (51.9)	*χ^2^_(1)_* = 0.35	0.554	*Cramer’s V = *0.058
Ongoing psychotherapy status		19 (36.5)	16 (30.8)	*χ^2^_(1)_* = 0.39	0.534	*Cramer’s V = *0.061
Medical comorbidity		4 (7.7)	7 (13.5)	*χ^2^_(1)_* = 0.92	0.339	*Cramer’s V = *0.094
Family history of psychiatric illness		22 (42.3)	22 (42.3)	*χ^2^_(1)_* <0.001	1.000	*Cramer’s V *<0.001
Income level	Low income	12 (23.1)	5 (9.6)	*χ^2^_(3)_* = 5.30	0.151	*Cramer’s V *= 0.226
	Lower-middle income	27 (51.9)	33 (63.5)			
	Upper-middle income	6 (11.5)	10 (19.2)			
	High income	7 (13.5)	4 (7.7)			

NSSI+, adolescents with non-suicidal self-injury; NSSI−, adolescents without non-suicidal self-injury; BMI, body mass index; M, mean; SD, standard deviation; *t*, independent samples *t*-test; *χ^2^*, chi-square test; f, female; m, male.

Among adolescents in the NSSI+ group, the most frequently reported self-injurious behaviors were cutting (98.1%), hitting oneself or striking against a hard surface (75%), interfering with wound healing (75%), scratching (69.2%), and pinching (63.5%). Additional behaviors included hair pulling (57.7%), skin scraping (48.1%), needle sticking (48.1%), biting (46.2%), burning (30.8%), and rubbing the skin against a hard surface (30.8%).

A MANOVA examining differences in CDI, PAS, SHAPS, and beta-endorphin levels across these groups detected a significant overall multivariate effect of group (Pillai’s Trace = 0.483, *F*(4, 99) = 23.16, *p* < 0.001, Partial η^2^ = 0.483). Follow-up univariate analyses indicated that adolescents in the NSSI+ group had significantly higher CDI, PAS, and SHAPS scores as well as significantly lower serum beta-endorphin levels when compared with those in the NSSI− group (Table 2).

**Table 2.** **Clinical and biological characteristics of the study groups (n = 104)**.

	NSSI+ (n = 52), M ± SD	NSSI− (n = 52), M ± SD	*F*(1, 102)	*p*-value	Partial η^2^
CDI scores	33.73 ± 6.44	23.33 ± 5.69	76.19	<0.001	0.428
PAS scores	53.52 ± 8.49	39.40 ± 12.17	47.05	<0.001	0.316
SHAPS scores	5.96 ± 3.02	3.33 ± 2.72	21.83	<0.001	0.176
Beta-endorphin levels (pg/mL)	81.19 ± 35.93	108.27 ± 68.66	6.35	0.013	0.059

M, mean; SD, standard deviation; F, MANOVA; CDI, Children’s Depression Inventory; PAS, Psychache Scale; SHAPS, Snaith-Hamilton Pleasure Scale. Overall multivariate effect: Pillai’s Trace = 0.483, *F*(4, 99) = 23.16, *p* < 0.001, Partial η^2^ = 0.483.

**Fig. 1.**
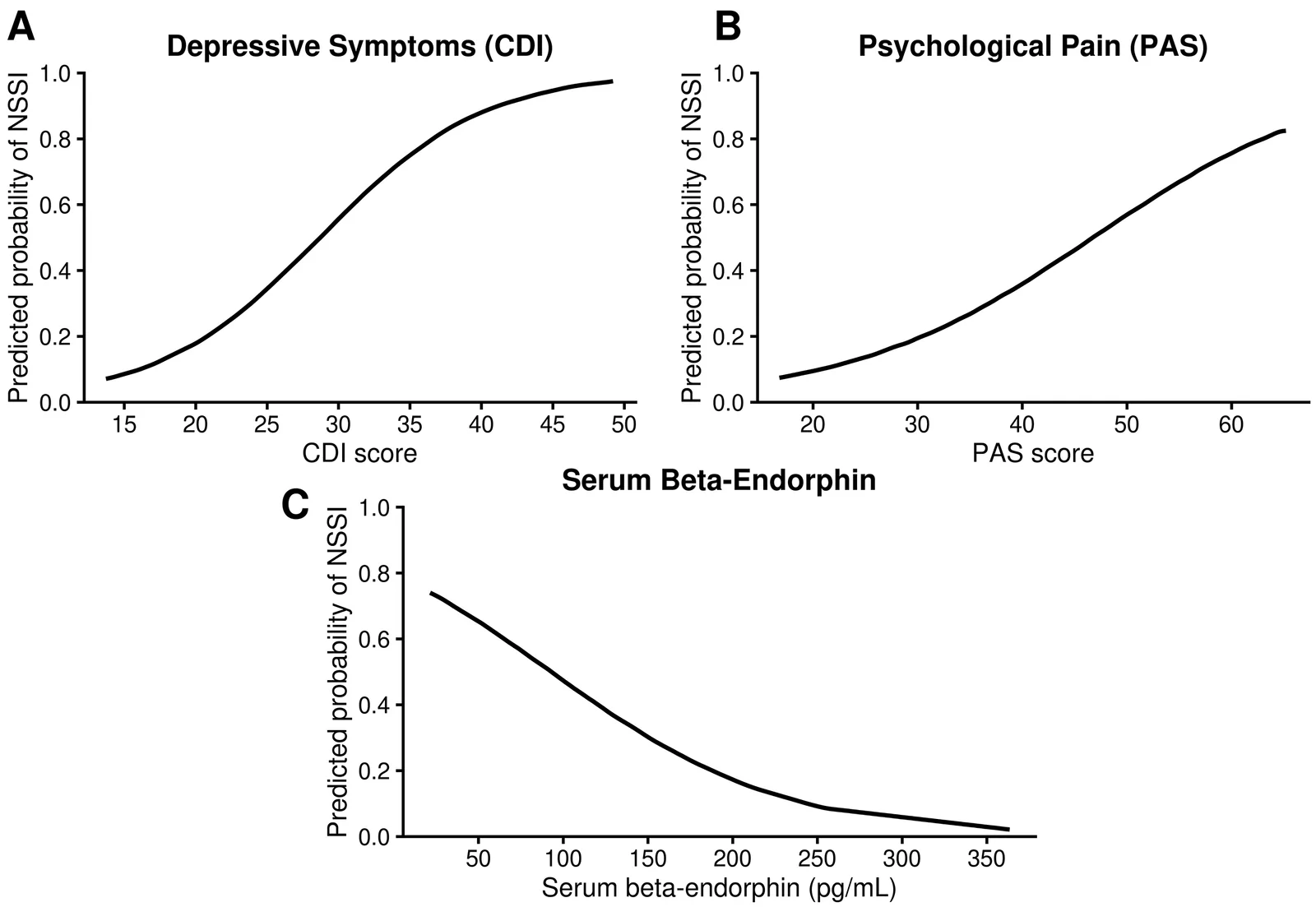
**Predicted probability of NSSI based on significant predictors from the logistic regression model**. (A) Depressive symptoms severity score. (B) Psychological pain (PAS) score. (C) Serum beta-endorphin level.

To examine factors associated with NSSI, a multivariable logistic regression analysis was conducted. The model demonstrated good fit (Hosmer–Lemeshow test, *p* = 0.230) and explained 62.6% of the variance in NSSI status in this cohort (Nagelkerke *R^2^* = 0.626). Multicollinearity diagnostics failed to detect any evidence of problematic collinearity among predictors (VIF = 1.064–2.493; tolerance = 0.401–0.940). Higher depressive symptom severity, greater psychological pain, and lower serum beta-endorphin levels were all found to be independently associated with patients having engaged in NSSI, whereas anhedonia was not a significant predictor of NSSI status (Table 3). Fig. 1 illustrates the predicted probability of NSSI according to the significant predictors identified in the logistic regression model. Materially similar results were also obtained from sensitivity analyses additionally controlling for age, sex, psychotropic medication use, psychotherapy status, and medical comorbidity, with no changes in the main findings. Lower beta-endorphin levels (OR = 0.980, 95% CI = 0.965–0.996, *p* = 0.012), higher depressive symptom severity (OR = 1.278, 95% CI = 1.097–1.488, *p* = 0.002), and greater psychological pain (OR = 1.082, 95% CI = 1.004–1.166, *p* = 0.040) remained significantly associated with NSSI, whereas anhedonia remained non-significant (**Supplementary Table 1**).

**Table 3.** **Logistic regression analysis predicting NSSI in adolescents with MDD (n = 104)**.

Independent variables	B	SE	OR	*p*-value	95% CI
CDI	0.176	0.060	1.193	0.003	[1.060–1.342]
PAS	0.085	0.035	1.089	0.014	[1.017–1.165]
Beta-endorphin	–0.015	0.007	0.985	0.030	[0.972–0.999]
SHAPS	0.079	0.117	1.082	0.500	[0.861–1.360]

MDD, major depressive disorder; CDI, Children’s Depression Inventory; PAS, Psychache Scale; SHAPS, Snaith-Hamilton Pleasure Scale; B, unstandardized beta coefficient; SE, standard error; OR, odds ratio; CI, confidence interval. Omnibus Test: *χ^2^* = 65.877, *p* < 0.001, Hosmer and Lemeshow: *p* = 0.230, Nagelkerke *R^2^* = 0.626.

**Fig. 2.**
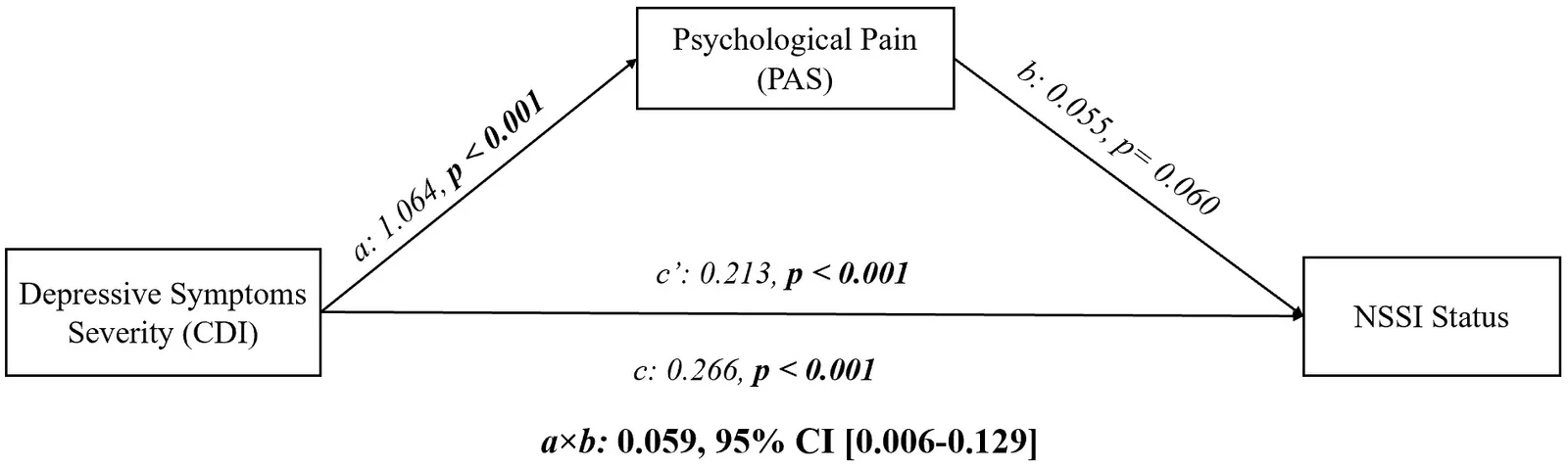
**Exploratory mediation model highlighting the role of psychological pain in the association between depression severity and NSSI (n = 104)**. This figure illustrates the exploratory indirect association between depression severity and NSSI through psychological pain. Path *a* represents the effect of depression on psychological pain (*p* < 0.001). Path *b* reflects the effect of psychological pain on NSSI after controlling for depression (*p* = 0.060). Path *c’* represents the direct effect of depression on NSSI after accounting for psychological pain (*p* < 0.001), whereas path *c* represents the total effect of depression on NSSI (*p* < 0.001). The indirect effect (*a* × *b*) was statistically significant, with a 95% bootstrap confidence interval (based on 5000 resamples) ranging from 0.006 to 0.129. Abbreviations: NSSI, non-suicidal self-injury; CI, confidence interval.

Finally, exploratory mediation analyses conducted in the full sample (n = 104) indicated a significant indirect association between depressive symptom severity and NSSI through psychological pain. Depression severity was significantly associated with psychological pain (*β* = 1.064, *p* < 0.001) and with NSSI *(β* = 0.213, *p *< 0.001). The indirect effect via psychological pain was significant (95% CI = 0.006–0.129), whereas the association between psychological pain and NSSI, after controlling for depressive symptom severity, was marginal (*β* = 0.055, *p* = 0.060) (Table 4; Fig. 2).

**Table 4.** **Exploratory examination of the indirect associations between depression severity, psychological pain, and NSSI in adolescents with MDD (n = 104)**.

Independent variables	Dependent variables					
	Psychological pain (M)			NSSI (Y)		
	*β*	*SE*	*p*	*β*	*SE*	*p*
Depressive symptoms severity (X)	1.064	0.116	<0.001	0.213	0.054	<0.001
Psychological pain (M)	-	-	-	0.055	0.029	0.060
Constant	16.109	3.421	<0.001	–8.614	1.638	<0.001
	*R^2^* = 0.454, *F*(1, 102) = 84.816, *p* < 0.001			*R^2^* (*Nagelkerke*) = 0.578, *p* < 0.001		

*β*, standardized beta coefficient; *SE*, standard error; X, independent variable; M, mediator variable; Y, dependent variable.

## 4. Discussion

In the present study, we examined biological and psychological correlates of NSSI in adolescents diagnosed with MDD. Through this approach, we found that adolescents with MDD who engage in NSSI exhibit significantly lower serum beta-endorphin levels, higher levels of psychological pain, and greater depressive symptom severity relative to those without NSSI. Exploratory analyses further suggested a significant indirect association between depressive symptom severity and NSSI through psychological pain, whereas anhedonia was not found to be an independent predictor of NSSI when depressive severity was taken into account. Taken together, these findings suggest that NSSI in adolescents with MDD may be more closely associated with emotional distress, psychological pain, and altered endogenous opioid functioning than with deficits in hedonic functioning alone.

Consistent with previous reports, adolescents with MDD and comorbid NSSI in the present study cohort exhibited significantly lower serum beta-endorphin levels compared to those without NSSI. Prior studies have observed reduced beta-endorphin concentrations in individuals engaging in self-injurious behaviors, although many of these investigations included diagnostically heterogeneous samples [12,13,26]. By focusing specifically on adolescents diagnosed with MDD, the present study extends the existing evidence and provides further support for a potential association between altered endogenous opioid functionality and NSSI within this clinical population. Under the opioid deficiency hypothesis, reduced endogenous opioid functioning is thought to increase vulnerability to engaging in self-harm by enhancing sensitivity to the affect-regulating effects of pain [8]. In line with this notion, previous research has demonstrated that beta-endorphin levels tend to be lower prior to episodes of self-injury and may transiently increase following self-harm [38]. Within this framework, a reduction in baseline beta-endorphin levels in adolescents with MDD may be associated with the maintenance of NSSI behaviors by contributing to the use of self-injurious behavior as a maladaptive strategy for modulating affective distress. Nevertheless, peripheral beta-endorphin levels cannot be considered a direct indicator of central opioid system functionality, and a degree of caution is thus needed when interpreting these findings.

Our findings further suggest that psychological pain may serve as an important factor associated with NSSI among adolescents with MDD. While psychological pain has been strongly associated with suicidality [39], its role in NSSI has received comparatively less empirical attention. Prior studies suggest that psychological pain may emerge from adverse developmental experiences—such as childhood trauma [40], invalidating environments [41], and insecure attachment patterns [42]—and may function as a proximal driver of self-injurious behavior. These findings also align with broader literature emphasizing the role of psychosocial vulnerability and emotional distress in self-injurious behaviors [43]. In this context, NSSI has been conceptualized as a maladaptive strategy aimed at alleviating overwhelming internal distress and restoring a sense of emotional control [17,44]. Consistent with this framework, our findings indicate that adolescents who engage in NSSI report significantly higher levels of psychological pain as compared to those without a history of self-harm. Moreover, exploratory analyses suggested a significant indirect association between depressive symptom severity and NSSI mediated through psychological pain, supporting the possibility that psychological pain may be linked to the relationship between depressive distress and self-injurious behavior. Psychological pain may therefore represent a clinically relevant construct that offers value as a means of understanding NSSI behaviors among adolescents with MDD. However, these findings should be interpreted with caution, given the inability to determine the temporal ordering of the examined variables and the marginal significance of the direct effect of psychological pain on NSSI.

The coexistence of lower beta-endorphin levels and elevated psychological pain in adolescents with NSSI may be indicative of some partial overlap in terms of neurobiological and affective processes. Prior review articles have emphasized the overlapping neural regions associated with psychological pain and physical pain, particularly the anterior cingulate cortex, prefrontal cortex, insula, and thalamus [24,45]. Experimental and neuroimaging studies have also suggested that psychological pain may be related to the function of the endogenous opioid system [46,47]. Within this framework, changes in endogenous opioid functionality may give rise to both heightened psychological pain and the affect-regulatory functions of self-injurious behavior.

Anhedonia has been consistently associated with suicidal ideation, independent of overall depression severity. A meta-analysis by Ducasse et al. (2018) [48] demonstrated that anhedonia remains a significant predictor of suicidal ideation even when controlling for the severity of depressive symptoms. Furthermore, individuals engaging in both NSSI and suicide attempts tend to exhibit higher levels of anhedonia compared to those engaging in NSSI alone [49,50]. However, the specific role of anhedonia in NSSI remains less clearly defined. In previous reports, anhedonia has been suggested to contribute to NSSI by reducing sensitivity to positive reinforcement and facilitating self-injurious behaviors as a maladaptive affect regulation strategy [23]. Longitudinal findings further indicate that although anhedonia may not directly mediate the association between depressive symptoms and NSSI, it still has the potential to influence individuals’ appraisal of NSSI as a functional coping strategy [51]. In line with this body of evidence, our findings indicate that adolescents with MDD who engage in NSSI report significantly higher levels of anhedonia. However, when depressive symptom severity is taken into account, anhedonia fails to independently predict NSSI, suggesting that its contribution may be secondary to broader affective dysregulation processes rather than a primary driver of self-harm in this adolescent population.

Taken together, these findings suggest that NSSI in adolescents with MDD is characterized by a constellation of affective and biological vulnerabilities, including elevated levels of psychological pain and aberrant endogenous opioid functionality. Rather than reflecting deficits in hedonic capacity alone, NSSI in this population appears to be more closely associated with the regulation of emotional distress. These findings highlight the importance of considering both psychological and biological perspectives when conceptualizing self-injurious behavior among depressed adolescents. Through additional longitudinal and mechanistic studies incorporating psychological, environmental, and biological factors, it may be possible to develop an increasingly robust understanding of the relationships between psychological pain, endogenous opioid function, and NSSI in adolescents with MDD, thereby contributing to a broader understanding of self-injurious behavior in this population.

### Limitations

There are certain limitations to this study that should be taken into consideration. First, because depressive symptoms, psychological pain, beta-endorphin levels, and NSSI were assessed contemporaneously, causal inferences regarding their temporal relationships cannot be drawn. Accordingly, the indirect association detected in this study should be treated as preliminary, given that temporal relationships between depressive symptoms, psychological pain, and NSSI could not be established. Longitudinal studies will be essential to address this by clarifying directionality and developmental trajectories. Second, serum beta-endorphin levels were assessed at a single time point, and thus may not fully capture the dynamic neurobiological processes associated with stress or self-injurious behavior. In addition, although no significant differences were observed between groups with respect to psychotropic medication use, psychotherapy status, or medical comorbidity, and sensitivity analyses controlling for these variables yielded materially similar findings, residual confounding related to unmeasured treatment characteristics (e.g., medication type, dosage, and duration) cannot be completely excluded. Third, the reliance of this study on self-report measures may have introduced reporting bias despite the use of validated instruments. Furthermore, psychological pain was assessed using a single self-report measure, with no corresponding comprehensive evaluation of the multidimensional aspects of psychological pain. Additionally, inter-rater reliability for NSSI classification was not formally evaluated, which may have affected the consistency of group assignment. Moreover, adolescents with subthreshold self-injurious behaviors were excluded from the NSSI− group; therefore, the findings may not be fully generalizable to adolescents with MDD who engage in occasional self-injurious behaviors that do not meet full DSM-5 criteria for NSSI. In addition, no formal correction for multiple comparisons was applied, potentially contributing to a greater risk of Type I error, such that the findings should be interpreted with caution. Fourth, relevant environmental, social, and psychosocial factors potentially associated with NSSI were not systematically evaluated, which may have influenced psychological pain, beta-endorphin levels, and NSSI, as well as the observed associations among these variables. Fifth, the absence of a healthy control group precludes any determination as to whether the observed findings are specific to NSSI within MDD patient cohorts or more broadly related to depression itself. Therefore, the findings should be interpreted as reflecting differences between adolescents with MDD with and without NSSI rather than disorder-specific alterations. Future studies including healthy controls may help clarify whether alterations in beta-endorphin levels, psychological pain, and anhedonia are uniquely associated with NSSI or reflect broader features of MDD. Sixth, the exploratory indirect association analyses performed herein should be interpreted cautiously, given the relatively modest sample size, underscoring the need for additional replication of these analyses in a larger cohort. Finally, the single-center design and relatively modest sample size may restrict broader generalization of the present findings, emphasizing the importance of conducting large-scale, multisite investigations.

## 5. Conclusions

The present study demonstrates that lower serum beta-endorphin levels, elevated levels of psychological pain, and greater depressive symptom severity are significantly associated with the presence of NSSI in adolescents with MDD, with the NSSI group meeting full DSM-5 diagnostic criteria for NSSI.

Exploratory analyses suggested a significant indirect association between depressive symptoms and NSSI through psychological pain, highlighting the potential relevance of psychological pain in the relationship between affective distress and self-injurious behavior. Nevertheless, the direct association between psychological pain and NSSI did not reach statistical significance. In contrast, anhedonia was not found to be an independent predictor of NSSI when depressive severity was taken into consideration. These findings highlight the importance of assessing both the biological and psychological dimensions of distress in adolescents with MDD and suggest that psychological pain and altered endogenous opioid functionality may be relevant to understanding the incidence of NSSI in this population.

## Data Availability

The data that support the findings of this study are available from the corresponding author upon reasonable request.
