# Supplementary material for: Non-Suicidal Self-Injury in Adolescents With Major Depressive Disorder: The Role of Beta-Endorphin, Psychological Pain, and Anhedonia
Source: Alpha Psychiatry. 2026 Aug 12;27(4):52361. doi: 10.31083/AP52361 (PMC13540034; doi:10.31083/AP52361)
Supplement: Supplementary file 1 [file 2757-8038-27-4-52361-s1.zip › Supplementary Table 1.pdf]

Supplementary Table S1. Sensitivity Analysis of the Logistic Regression Model Predicting NSSI After Adjustment for Demographic, Treatment-Related, and Clinical Covariates

Independent Variables	*B*	*SE*	*OR*	*p*	*95% CI*
Age	0.344	0.249	1.410	0.167	[0.866-2.298]
Gender (male, ref: female)	0.935	0.832	2.548	0.261	[0.499-13.002]
Current psychotropic medication use	0.775	0.621	2.170	0.212	[0.642-7.335]
Ongoing psychotherapy	0.887	0.663	2.427	0.181	[0.662-8.892]
Medical comorbidity	-1.017	1.219	0.362	0.404	[0.033-3.942]
CDI	0.245	0.078	1.278	0.002	[1.097-1.488]
PAS	0.079	0.038	1.082	0.040	[1.004-1.166]
Beta-endorphin (pg/mL)	-0.020	0.008	0.980	0.012	[0.965-0.996]
SHAPS	0.049	0.122	1.050	0.690	[0.827-1.333]

†**CDI:** Children's Depression Inventory, **PAS:** Psychache Scale, **SHAPS:** Snaith-Hamilton Pleasure Scale **B:** Unstandardized Beta Coefficient, **SE:** Standard Error, **OR:** Odds Ratio, **CI:** Confidence Interval ‡Omnibus Test: χ^2=71.873, $p < 0.001$, Hosmer and Lemeshow: $p = 0.759$, Nagelkerke $R^2 = 0.665$
